# Validation of reference genes for gene expression analysis in olive (*Olea europaea*) mesocarp tissue by quantitative real-time RT-PCR

**DOI:** 10.1186/1756-0500-7-304

**Published:** 2014-05-18

**Authors:** Debashree L Ray, Joshua C Johnson

**Affiliations:** 1 College of Engineering & Science, Victoria University, PO Box 14428, Melbourne, VIC 8001, Australia; 2 Institute for Sustainability & Innovation, Victoria University, PO Box 14428, Melbourne, VIC 8001, Australia

**Keywords:** Reference genes, qRT-PCR, *Olea europaea*, Gene expression

## Abstract

**Background:**

Gene expression analysis using quantitative reverse transcription PCR (qRT-PCR) is a robust method wherein the expression levels of target genes are normalised using internal control genes, known as reference genes, to derive changes in gene expression levels. Although reference genes have recently been suggested for olive tissues, combined/independent analysis on different cultivars has not yet been tested. Therefore, an assessment of reference genes was required to validate the recent findings and select stably expressed genes across different olive cultivars.

**Results:**

A total of eight candidate reference genes [glyceraldehyde 3-phosphate dehydrogenase (*GAPDH*)*,* serine/threonine-protein phosphatase catalytic subunit (*PP2A*)*,* elongation factor 1 alpha (*EF1-alpha*)*,* polyubiquitin (*OUB2*)*,* aquaporin tonoplast intrinsic protein (*TIP2*)*,* tubulin alpha (*TUBA*)*,* 60S ribosomal protein L18-3 (*60S RBP L18-3*) and polypyrimidine tract-binding protein homolog 3 (*PTB*)] were chosen based on their stability in olive tissues as well as in other plants. Expression stability was examined by qRT-PCR across 12 biological samples, representing mesocarp tissues at various developmental stages in three different olive cultivars, Barnea, Frantoio and Picual, independently and together during the 2009 season with two software programs, GeNorm and BestKeeper. Both software packages identified *GAPDH*, *EF1-alpha* and *PP2A* as the three most stable reference genes across the three cultivars and in the cultivar, Barnea. *GAPDH*, *EF1-alpha* and *60S RBP L18-3* were found to be most stable reference genes in the cultivar Frantoio while *60S RBP L18-3, OUB2* and *PP2A* were found to be most stable reference genes in the cultivar Picual.

**Conclusions:**

The analyses of expression stability of reference genes using qRT-PCR revealed that *GAPDH*, *EF1-alpha*, *PP2A, 60S RBP L18-3* and *OUB2* are suitable reference genes for expression analysis in developing *Olea europaea* mesocarp tissues, displaying the highest level of expression stability across three different olive cultivars, Barnea, Frantoio and Picual, however the combination of the three most stable reference genes do vary amongst individual cultivars. This study will provide guidance to other researchers to select reference genes for normalization against target genes by qPCR across tissues obtained from the mesocarp region of the olive fruit in the cultivars, Barnea, Frantoio and Picual.

## Background

Quantitative reverse transcription PCR (qRT-PCR) is a well-established procedure to study changes in gene expression levels, due to its high sensitivity, reproducibility and large dynamic range [1-6]. In qRT-PCR experiments using a relative quantification approach, the expression level of the target genes are normalised using internal control genes known as reference genes to derive changes in gene expression levels. This normalisation strategy improves the fidelity of the quantification process by controlling any variation in the expression level of the biological samples that might have been introduced due to various factors such as RNA integrity, initial sample amount, reverse transcription efficiency etc. Some of the most common and best known housekeeping genes involved in basic cellular and metabolic processes that have been used as candidate reference genes over the last few decades in plants and animals include *GAPDH*, *18S* or *26S RNA*, *EF1-alpha*, *ubiquitin carrier protein*, *actin, α-tubulin, β-tubulin* and *TATA-Box binding protein*[3,5,7,8]. It is assumed that these reference genes have constant level of expression in different tissues and under different treatments and has no inter-individual variability [9,10]. Experimentally, it is impossible to find a single, ideal reference gene for normalisation in various samples, under different conditions as the transcription of any gene will not be absolutely resistant to fluctuations in the cell cycle or nutrition status [5,7]. It has been shown that in many experiments the use of a single reference is not acceptable as it would likely produce erroneous conclusions in expression patterns [11,12]. Recent reports have also shown that the most commonly used traditional reference genes may be inappropriate for normalization in qPCR experiments due to their expression variability under different experimental conditions [8,13]. The importance of expression stability in the choice of reference genes has prompted the development of various software packages including GeNorm [4], BestKeeper [14] and NormFinder [15] to identify such genes. Therefore, for accurate analysis of RNA transcription it is crucial to choose reference genes that have been shown to be minimally regulated in a given species, in a given organs/tissues, at a given developmental period and under specific environmental conditions.

In plants a number of reference gene validation reports have been published covering both model and agriculturally important crop species including, *Arabidopsis*[8], rice [16], wheat [5] grapevine [3], barley [7], soybean [9] and cotton [10]. In *Arabidopsis thaliana*, analysis of the Affymetrix ATH1 microarray data has revealed that there are hundreds of potential reference genes that outperform traditional reference genes in terms of expression stability, where most of these genes are expressed at much lower levels than the traditional reference genes. This list of new *Arabidopsis* reference genes were successfully employed to search for reference genes in unrelated species such as *Vitis vinifera*[17] and *Coffea arabica*[18].

The olive tree (*Olea europaea*) represents one of the oldest agricultural tree crops with cultivation of olives beginning in the Mediterranean basin more than 3000 years ago [19]. Over the past few decades an increased awareness of the reported health benefits of olive oil over other fats and oils [20-24] has seen olive oil consumption increase globally [19,25] and cultivation of olives has spread globally into new areas such as Australia, North America and South America [19]. In the past few years high throughput next generation sequencing (NGS) technologies have been used to study the transcriptome of olive drupes during different developmental stages and tissues [26-28]. These sequencing data are a valuable resource for gene discovery and characterisation in olives. However, in order to normalise the expression data of genes in olives it is important to identify stable reference genes which show consistent expression within olive samples for a specific set of chosen experimental conditions and tissue types in olives.

In olives, an analysis of cDNAs that are associated with alternate bearing in olive was conducted wherein seven commonly used reference genes [*Olest34*, *alpha-tubulin*, *beta-tubulin*, *beta-actin*, *26S rRNA*, *18S rRNA* and *GAPDH*] were chosen to identify the reference gene which is least spatially and temporally variable [28]. *GAPDH* was decided to be used as an appropriate reference gene for the major tissues (leaves, fruits and pedicels) of olive to normalise the copy numbers of the cDNAs tested [28]. Although the authors did provide data to justify this selection there was not an in-depth analysis of potential reference gene candidates.

More recently, two studies were conducted to identify stable reference genes in olives during fruit development and ripening. Nonis *et al.*[29] focused on the stability of 13 putative reference genes evaluated on 21 samples collected over different developmental stages of olive fruits and leaves subjected to wounding from the cultivar Frantoio during the 2009 season. Thirteen candidate reference genes belonging to 8 gene families were chosen based on their stability in other plants as well as the availabilty of putative ESTs for these genes in a publicly available database of olive ESTs [26]. Results showed that serine/threonine-protein phosphatase catalytic subunit-1 (*PP2A1*) was the most transcriptionally stable reference gene, followed by *GAPDH2*, while *GAPDH1* showed the widest variation and was considered the least stable gene. Another study was conducted on the validation analysis of 29 reference genes at 12 different sampling points of olive fruit development from the cultivar, Istrska belica using two evaluation approaches, GeNorm and RefFinder programs [30]. Combining both the programs together, TIP41-like family protein *(TIP41)* and TATA binding protein *(TBP)* were identified as the two most stable reference genes during fruit development in this cultivar.

Comparing the results from the two papers [29,30], it is interesting to note that few reference genes such as *EF1-alpha, GAPDH, 14-3-3 protein* performed differently in these two experiments during the olive fruit development with differences in their M-values and ranking order clearly indicating that the stability of the reference genes must be validated with each experimental setup. It is to be noted that though both the studies identified stable reference genes during olive fruit development and ripening, these experiments were conducted on different olive cultivars, with the former study conducted on the cultivar Frantoio while the latter was on the cultivar Istrska belica. This indicates that even for different cultivars the stability of the reference genes should be assessed.

Thus, the current study involved the use of two software packages, GeNorm (qBase Plus) and BestKeeper, to validate the expression stability of eight candidate reference genes chosen based on their stability in olive tissues as well as in other plants, during mesocarp development across three different olive cultivars Barnea, Frantoio and Picual independently and together during the 2009 season by qPCR.

## Results

### Assessment of RNA integrity and verification of PCR products

Agarose gel electrophoresis images of total RNA extracted from all olive samples revealed two distinct bands representing 18S- and 28S- RNA bands (data not shown) and the RNA integrity number (RIN) values for all samples ranged from 8.3-9.9 (data not shown). Amplification of the eight candidate reference genes by PCR revealed products of the expected sizes (Additional file 1) and subsequent sequencing of these products revealed that all amplified fragments were identical to sequences used for designing primers for the reference genes (Table 1) (sequencing data not shown).

**Table 1:** **Eight candidate reference genes assessed for gene expression normalisation in *Olea europaea *and amplicon characteristics**

**Gene name**	Accession number/cluster ID*	**Primer sequence (5' to 3')**	**Amplicon length (bp)**	Annealing temperature** (°C)	PCR efficiency value***	**Standard error (SE)**	**R**^2 ^****
*60S RBP L18-3*	[Oleadatabase:Cluster ID-OLEEUCl011221] contig 2	F: GTAAGAGCAAGAAGACCAAG	101	55	1.984	0.014	0.978
		R: GCTTCCAGTTCTCCTCAC					
*PP2A*	[Oleadatabase:Cluster ID-OLEEUCl010038] contig 1	F: AGATCGGTGAAATACTTCCACACG	189	56	2.038	0.020	0.969
		R: TCGTGGATACTACTCAGTGGAGACTG					
*PTB*	[Oleadatabase:Cluster ID-OLEEUCl031691] contig 1	F: CTTCTCCGAAATAAACCAGAT	156	56	2.243	0.057	0.855
		R: GGTGTCAGCTCCAGTTGTAA					
*TUBA*	[Oleadatabase:Cluster ID-OLEEUCl051890] contig 1	F: AGAACACCTCAGCAACAC	100	51	1.704	0.069	0.870
		R: AACTACCAGCCACCAACT					
*TIP2*	[Oleadatabase:Cluster ID-OLEEUCl011159] contig 2	F: ACTTGTTGTAAGCAATGG	104	51	2.093	0.081	0.935
		R: TGATTCATTAAGCGTTGG					
*OUB2*	[GenBank:AF429430.1]	F: AATGAAGTCTGTGTGTCCTTTGG	150	51	1.513	0.054	0.890
		R: AAGGGAAATCCCATCAACG					
*GAPDH*	[GenBank:EF506530.1]	F: ACAGCTCCTGGTAAGGGTGA	210	56	2.111	0.018	0.971
		R: GGCTTGCGTCAAGAAGTCTC					
*EF1-alpha*	[GenBank:XM_002527974.1]	F: GAATGGTGATGCTGGTTTCG	191	56	1.908	0.005	0.996
		R: CCCTTCTTGGCAGCAGACTTG					

*Cluster ID obtained from the *Olea* database [26].

**Annealing temperature used in PCR experiments.

***Measure of the PCR amplification efficiency calculated from calibration curve derived from prepared standards.

****correlation coefficient (R^2^) for the calibration curve.

### Determination of PCR amplification efficiency and melting curve analysis

The amplification efficiencies (E) for the eight candidate reference genes ranged between 1.513-2.243, standard errors (SD) between 0.005-0.081 and the R^2^ value between 85.5-99.6% (Table 1) (Additional file 2). Amplification of all reference genes revealed the presence of a single peak in the melt curve analysis except TIP2 which showed the presence of two peaks, a large peak at 83°C and a smaller peak at a lower temperature (~78.5°C) (Additional file 3). No signal was detected in the negative controls for all eight reference genes.

### Expression levels of candidate reference genes

**Figure 1:**
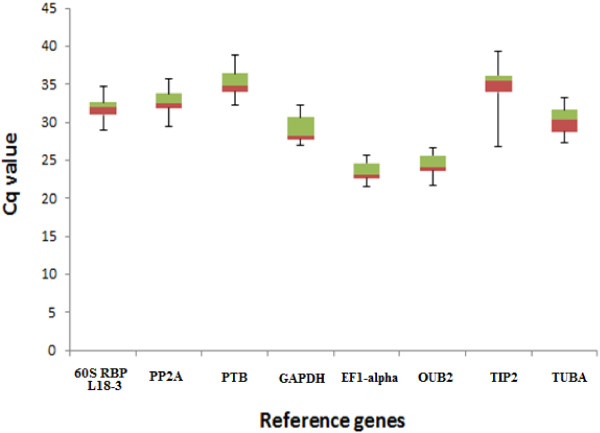
**Expression levels of eight candidate reference genes.** The values are given as real-time PCR quantification cycle (Cq) values for individual reference genes in a total of 12 samples (in duplicate) from the individual timepoints in the 2009 (96, 109, 116 and 136 DAF) crop season from cultivars Barnea, Frantoio and Picual. The boxes represent the 25^th^ and 75^th^ percentiles and the line within the boxes represents the median. The whiskers indicate the range of Cq values of the data of the 24 samples per reference gene.

The eight reference genes displayed a wide expression range with quantification cycle (Cq) values ranging from 21 to 39 (Figure 1) (Additional file 4). Highly expressed genes with Cq values between 21-25 cycles were *EF1-alpha* and *OUB2*. Genes with intermediate expression levels with Cq values ranging from 28-32 cycles were *GAPDH*, *PP2A*, *60S RBP L18-3* and *TUBA*. Genes with lower expression levels with Cq values >34 cycles were *PTB* and *TIP2*.

### Stability assessment of reference genes

The ranking of the eight candidate reference genes according to their expression stability across the three olive cultivars Barnea, Frantoio and Picual together and as independent cultivars as calculated by the geNorm are shown in Table 2.

**Table 2:** Ranking of the eight candidate reference genes according to their expression stability in two different algorithms GeNorm and BestKeeper in Barnea, Frantoio and Picual individually and together

	**Barnea + Frantoio + Picual**		**Barnea**		**Frantoio**		**Picual**	
**Ranking**	**GeNorm**	**BestKeeper**	**GeNorm**	**BestKeeper**	**GeNorm**	**BestKeeper**	**GeNorm**	**BestKeeper**
1	*GAPDH*	*PP2A*	*EF1-Alpha*	*PP2A*	*EF1-Alpha*	*GAPDH*	*60S RBP L18-3*	*60S RBP L18-3*
2	*EF1-Alpha*	*EF1-Alpha*	*GAPDH*	*EF1-Alpha*	*60S RBP L18-3*	*EF1-Alpha*	*PP2A*	*OUB2*
3	*PP2A*	*GAPDH*	*PP2A*	*GAPDH*	*PTB*	*OUB2*	*OUB2*	*PP2A*
4	*60S RBP L18-3*	*PTB*	*OUB2*	*OUB2*	*OUB2*	*60S RBP L18-3*	*EF1-Alpha*	*EF1-Alpha*
5	*PTB*	*60S RBP L18-3*	*TUBA*	*60S RBP L18-3*	*GAPDH*	*PTB*	*GAPDH*	*GAPDH*
6	*OUB2*	*TIP2*	*60S RBP L18-3*	*PTB*	*PP2A*	*PP2A*	*TUBA*	*PTB*
7	*TUBA*	*OUB2*	*PTB*	*TIP2*	*TUBA*	*TUBA*	*PTB*	*TIP2*
8	*TIP2*	*TUBA*	*TIP2*	*TUBA*	*TIP2*	*TIP2*	*TIP2*	*TUBA*

**Figure 2:**
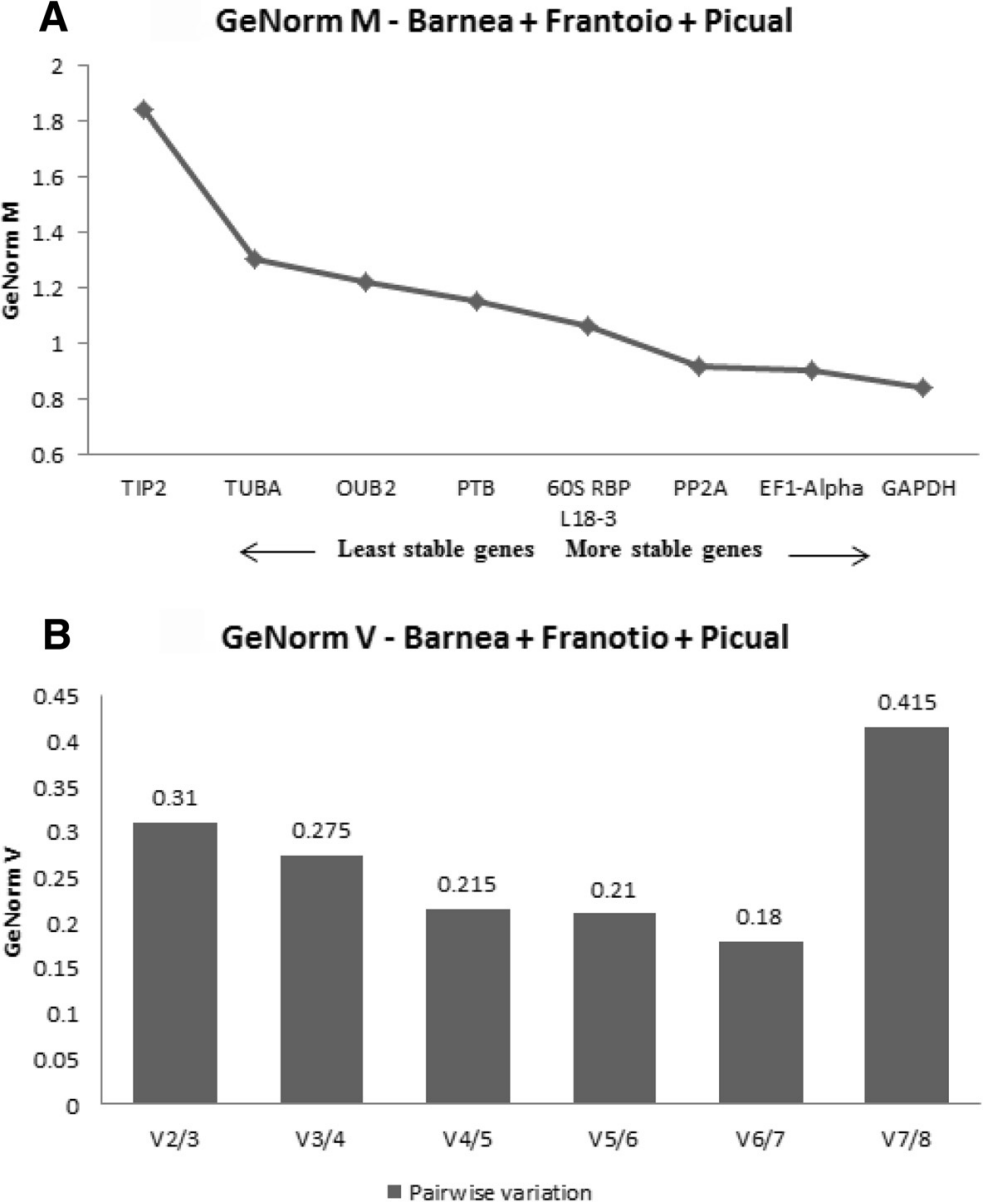
**Validation of candidate reference genes in Barnea, Frantoio and Picual as a whole using GeNorm algorithm in qBase Plus. (A)** Average expression stability values (M) of the eight reference genes plotted from least stable (left) to most stable (right). The M value was calculated for each gene and the least stable gene with the highest M value was excluded from the next calculation round. **(B)** Pairwise variation analysis (V_n_/V_n+1_) between the normalisation factors NFn and NFn + 1 to determine the optimal number of reference genes to be used for normalisation against target genes. GeNorm V calculates the normalisation factor (NF_n_) by calculating the geometric mean of the expression levels of the stable most reference genes by step-wise inclusion of a less stable gene.

The combined analysis across the three cultivars showed that the expression stability (M) of the eight reference genes studied varied dramatically with values ranging from 1.85 to 0.85 (Figure 2A). *TIP2* was the least stable gene with an M value of 1.85, whereas *GAPDH* was identified as the most stable gene, with an M value of 0.85 (Figure 2A). In addition to this, pairwise variation V_n_/V_n+1_ between two sequential normalisation factors NF_n_ and NF_n+1_ was also calculated to determine the optimal number of reference genes to be used for normalisation. According to GeNorm V, V6/7 showed the lowest pairwise variation of 0.18 (Figure 2B) indicating that six reference genes with the lowest M values were the optimal number of reference genes which should be used for the most accurate normalisation.

**Figure 3:**
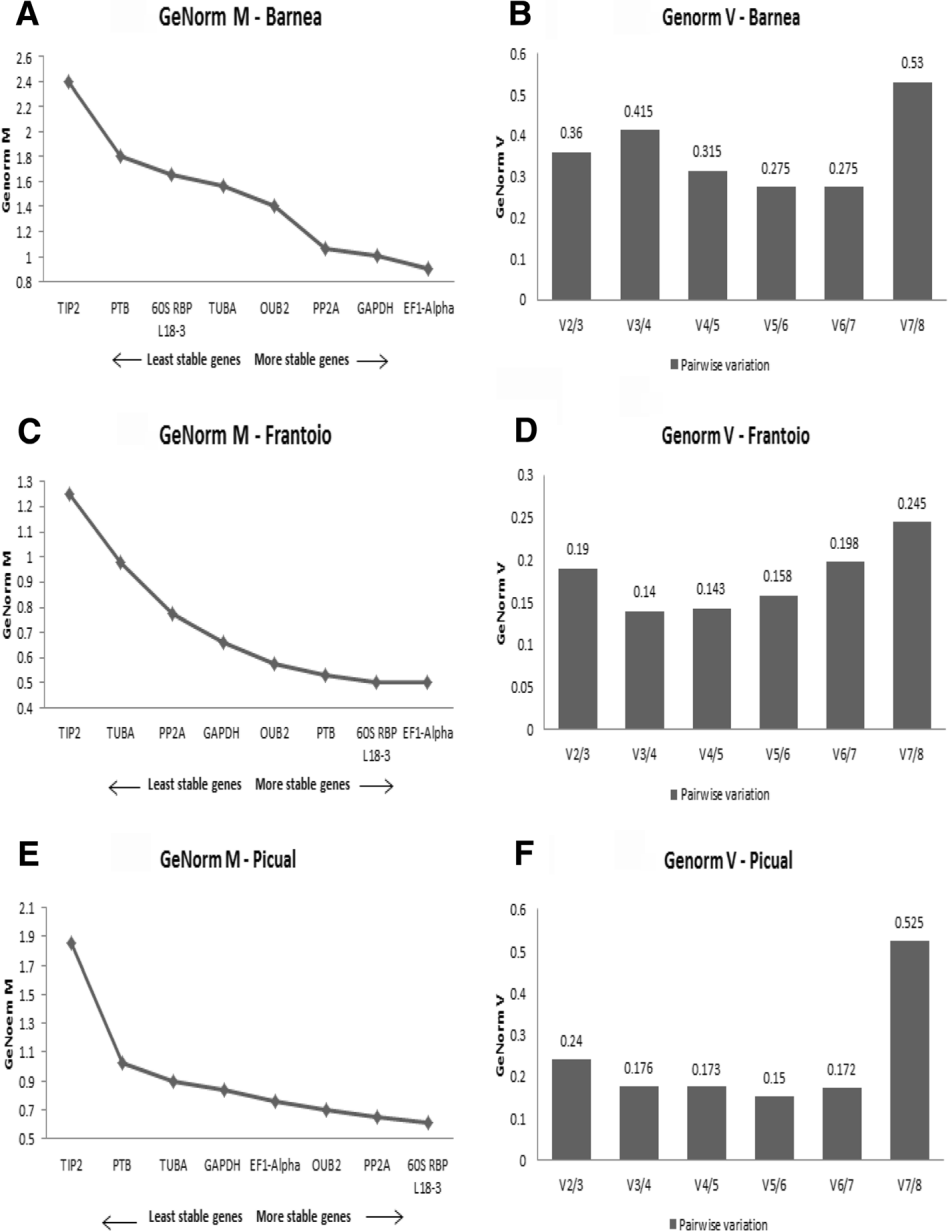
**Validation of candidate reference genes in Barnea, Frantoio and Picual independently using GeNorm algorithm in qBase Plus. (A)** Average expression stability values (M) of the eight reference genes plotted from least stable (left) to most stable (right) in Barnea **(A)**, Frantoio **(C)** and Picual **(E) (B)** Pairwise variation analysis (V_n_/V_n+1_) between the normalisation factors NFn and NFn + 1 to determine the optimal number of reference genes to be used for normalisation against target genesin Barnea **(B)**, Frantoio **(D)** and Picual **(F)**.

GeNorm was further used to evaluate the expression stability of the eight reference genes in each cultivar independently. In Barnea, the M values ranged from 0.9 to 2.4 where, *TIP2* was the least stable gene with an M value of 2.4, whereas *EF1-alpha* was identified as the most stable gene, with an M value of 0.9 (Figure 3A). According to GeNorm V, V5/6 showed the lowest pairwise variation of 0.275 (Figure 3B). In Frantoio, the M values ranged from 0.5 to 1.25 where, *TIP2* was the least stable gene with an M value of 1.25, whereas *EF1-alpha* was identified as the most stable gene, with an M value of 0.5 (Figure 3C). According to GeNorm V, V3/4 showed the lowest pairwise variation of 0.14 (Figure 3D). In Picual, the M values ranged from 0.61 to 1.85 where, *TIP2* was the least stable gene with an M value of 1.85, whereas *60S RBP L18-3* was identified as the most stable gene, with an M value of 0.61 (Figure 3E). According to GeNorm V, V5/6 showed the lowest pairwise variation of 0.15 (Figure 3F).

BestKeeper was also used to calculate and compare the gene expression variation for the eight candidate reference genes based on the geometric mean of their Cq values and displayed as the standard deviation (SD) and coefficient of variance (CV) (Table 3). The variation in gene expression of two reference genes *TIP2* and *TUBA* was greater than two-fold (SD greater than 1) in all three varieties individually and together. *OUB2* also had SD greater than 1 in the combined analysis of the three cultivars. The other reference genes had SD ≤ 1 and thus are considered to be stably expressed (p ≤ 0.05). The ranking of these reference genes was based on their pairwise correlation with the BestKeeper index value which is indicated by the Pearson correlation coefficient (r). In the combined analysis of the three cultivars (Tables 2 and 3A), BestKeeper recommended *PP2A* as the most stable gene with a correlation coefficient of 0.805. The comparison of the five other candidate reference genes with this r value resulted in a ranking as follows, from the least stable to the most stable: *60S RBP L18-3* > *PTB* > *GAPDH* > *EFI-alpha* > *PP2A*. In Barnea, *PP2A* was identified as the most stable gene with a correlation coefficient of 0.886 which was followed by *EF1-alpha, GAPDH, OUB2, 60S RBP L18-3* and *PTB* (Tables 2 and 3B)*.* In Frantoio, *GAPDH* was identified as the most stable gene with a correlation coefficient of 0.835 which was followed by *EF1-alpha, OUB2, 60S RBP L18-3, PTB* and *PP2A* (Tables 2 and 3C)*.* In Picual, *60S RBP L18-3* was identified as the most stable gene with a correlation coefficient of 0.969 which was followed by *OUB2, PP2A, EF1-alpha, GAPDH* and *PTB* (Tables 2 and 3D)*.*

**Table 3:** BestKeeper descriptive statistical analyses of eight reference genes in Barnea, Frantoio and Picual mesocarp together (A) and individually (B, C and D)

**Factors**	**Stability of reference genes in Barnea, Frantoio and Picual (A)**							
	***60S RBP L18-3***	***PP2A***	***PTB***	***GAPDH***	***EF1-alpha***	***OUB2***	***TIP2***	***TUBA***
n	24	24	24	24	24	24	24	24
SD [±Cq]	0.9	1	0.93	0.94	0.99	**1.07**	**2.64**	**1.54**
CV [% Cq]	2.87	3.08	2.69	3.29	4.26	4.41	7.66	5.11
r value	0.654	**0.805**	0.683	0.717	0.769	0.711	0.832	0.665
p value	0.001	0.001	0.001	0.001	0.001	0.001	0.001	0.001
Ranking	5	1	4	3	2	7	6	8
	**Stability of reference genes in Barnea (B)**							
n	8	8	8	8	8	8	8	8
SD [±Cq]	1.51	0.94	1.56	0.99	0.98	1.00	**3.50**	**1.50**
CV [% Cq]	4.86	3.49	4.56	4.31	4.29	4.45	10.12	4.91
r value	0.744	**0.886**	0.720	0.796	0.854	0.771	0.866	0.811
p value	0.034	0.003	0.044	0.039	0.007	0.023	0.005	0.014
Ranking	5	1	6	3	2	4	7	8
	**Stability of reference genes in Frantoio (C)**							
n	8	8	8	8	8	8	8	8
SD [±Cq]	0.53	0.47	0.26	0.68	0.44	0.28	**1.15**	**1.38**
CV [% Cq]	1.68	1.46	0.75	2.36	1.95	1.19	3.32	4.56
r value	0.778	0.686	0.713	**0.835**	0.827	0.796	0.510	0.682
p value	0.004	0.021	0.032	0.010	0.004	0.020	0.003	0.002
Ranking	4	6	5	1	2	3	8	7
	**Stability of reference genes in Picual (D)**							
n	8	8	8	8	8	8	8	8
SD [±Cq]	0.36	0.53	0.83	0.98	0.99	0.96	**3.32**	**1.51**
CV [% Cq]	1.13	1.64	2.38	2.56	2.80	2.42	9.66	5.04
r value	**0.969**	0.883	0.659	0.797	0.809	0.946	0.867	0.753
p value	0.001	0.004	0.015	0.018	0.015	0.001	0.005	0.031
Ranking	1	3	6	5	4	2	7	8

*Abbreviations*: *n* number of samples, *SD [±Cq]* standard deviation of Cq values, *CV [% Cq]* coefficient of variance expressed as percentage of Cq values, *r* coefficient of correlation, *p* probability value. Genes showing the highest r value and genes with SD > 1 have been highlighted in bold. Ranking of the eight reference genes based on their r-value and SD values have also been shown.

## Discussion

This is one of the first reports conducted to assess the expression stability of candidate reference genes at different developmental stages of the olive mesocarp and the first to assess the stability of these genes across different cultivars. For an accurate comparison of mRNA levels in different samples, it is crucial to normalize the expression of target genes against appropriate reference genes. An ideal reference gene should be expressed at constant level in all types of cells, at any time in cell cycle and differentiation and/or with any sample treatment [9,10,31]. Traditional reference genes such as *EF1-alpha* and *OUB2* (involved in basic cellular processes) or those encoding actin and tubulin (involved in cell structure maintenance) have been frequently used in qPCR experiments [3,5,7,8]. However research has shown that these genes may be inappropriate for normalization in qPCR experiments due to their expression variability under different experimental conditions, confirming the need to validate expression stability of reference genes in given species and organs/tissues under specific experimental conditions [8,13]. Normalization with multiple reference genes has become a gold standard in qPCR expression analysis [3,9,10] and also a requisite according to MIQE (Minimum Information for Publication of Quantitative Real-Time PCR Experiments) guidelines [2]. To this end, a number of software packages have been developed to assess the stability of candidate reference genes in different biological experimental settings, including, GeNorm, BestKeeper and NormFinder. However, the validation of reference genes is not very common in plant research [10,16,18].

Recently two studies were conducted to identify stable reference genes in olives during fruit development and ripening [29,30]. Although stable reference genes were suggested for the olive fruit developmental stages, a few reference genes, such as *EF1-alpha, GAPDH*, 14-3-3 protein performed differently in these two experiments during the olive fruit development with differences in their M-values and ranking order. These differences clearly show that the stability of the reference genes must be validated with each experimental setup. As two different cultivars were used in these two studies it is possible that differences in cultivars might explain these differences in gene expression between the reference genes indicating that even for different cultivars the stability of the reference genes should be assessed. Therefore, in this study a combination of three different olive cultivars Barnea, Frantoio and Picual were assessed both individually and together to evaluate the expression stability of eight candidate reference genes during olive mesocarp development.

The primers that were designed for the eight reference genes amplified single PCR products of the expected size from the olive cDNA pools as shown by gel electrophoresis (Additional file 1) and melt-curve analysis (Additional file 3) suggested that single products were amplified and that only TIP2 showed the formation of some primer-dimers. This specificity was confirmed by sequencing all eight PCR products which confirmed their identity to sequences used for designing primers for the reference genes.

Previous research has shown that analysis of expression stability of reference genes using different combinations of GeNorm, BestKeeper and NormFinder can result in minor to substantial discrepancies in the final ranking of the suitable reference genes which is typically explained by the differences in the mathematical models associated with each program [3,17]. In this study the results obtained by the two algorithms did not show much discrepancy and both the programs were compared for the final choice of suitable reference genes. The ranking of the eight candidate reference genes based on their stability (M) and correlation coefficient values as calculated using GeNorm (qBase Plus) and BestKeeper algorithms respectively across the three cultivars together and as individual cultivars are shown in Tables 2 and 3. According to GeNorm analysis in the three cultivars together, the two traditional reference genes *GAPDH* and *EF1-alpha* were the most stable with lowest M-values, followed by the reference gene *PP2A*, *60S RBP L18-3* and *PTB* were placed in the middle of the ranking, while *OUB2*, *TUBA* and *TIP2* displayed inappropriate expression stability with higher M-values and thus appear to be regulated in these tissues.

According to BestKeeper analysis of the three cultivars together, *PP2A* was ranked as the most suitable reference gene with the highest correlation coefficient of 0.805, followed by *EF1-alpha* and *GAPDH. PTB* and *60S RBP* were placed in the middle of the ranking, while *OUB2*, *TUBA* and *TIP2* showed SD ≥1 and thus were considered inconsistent. Although *TIP2* has the highest correlation coefficient of 0.832, it cannot be used for normalisation because of its unacceptable SD value (SD > 1).

Thus, *GAPDH*, *EF1-alpha* and *PP2A* were determined to be the three most stable reference genes analysed across the three cultivars when analysed together, albeit with slightly different rankings using the different analytical approaches. Many studies with similar findings, wherein different software packages identify the same reference genes as the most stable genes but not in the exact same ranking order have been reported in both animals and plants [31,32].

These findings are in broad agreement with the Nonis *et al*. [29] data which showed that *PP2A1* was the most transcriptionally stable reference gene during olive fruit development, followed by *GAPDH2,* while *GAPDH1* showed the widest variation and was considered the least stable gene. BLASTx of the *GAPDH* contig [GenBank:EF506530.1] used in this study showed that it is 97% identical to *Glycine max* GAPDH Subunit A [GenBank:NP_001238484.1]. BLASTx of the two different GAPDH transcripts used by Nonis *et al*[29] showed that [OLEEUCl022518|Contig2] and [OLEEUCl004899|Contig2] are 89-90% identical to *Glycine max* GAPDH Subunit C [GenBank: NP_001237544.1]. The PP2A contig [OLEEUCl010038 contig1] used in this study is 98% identical to *Glycine max* PP2A-2 [GenBank: XP_003532043.1]. BLASTx of the two different PP2A transcripts used by Nonis *et al*[29] showed that [OLEEUCl021775|Contig2] is 92% identical to *Glycine max* PP2A-3 [GenBank:XP_003533904.1] and [OLEEUCl021848|Contig 2] is 97% identical to *Glycine max* PP2A-1 [GenBank: XP_003534171.1]. Both *PP2A* transcripts used by Nonis *et al*[29] performed well in that study [29]. These studies clearly suggests that the *PP2A* gene family is stably expressed in olive fruit developmental stages. In addition, *PP2A* was also identified as a suitable reference gene in cotton across different plant organs [10] as well as in *Arabidopsis*[8]. *GAPDH*, a traditional reference gene, was considered the most suitable reference gene in coffee leaves under drought-stress and in different cultivars [18], however performed poorly across tissues and organs of tomato at different developmental stages [33]. *EF1-alpha* was found to be stably expressed in olive fruit developmental stages in two different studies, however with differences in M-values and ranking orders [29,30]. Under conditions of biotic and abiotic stress, *EF1-alpha* was found to be very stably expressed in potato [34], while *EF1-beta* was found to be the most stable in soybean [35]. *EF1-alpha* was also found to be stable in expression across different tissues of rice [16].

*PTB* and *60S RBP L18-3* were placed in the middle of the ranking order across the three cultivars as a whole by both software packages, again differing in their order (Table 2). *PTB* was identified as one of the most stable reference gene in cotton during fruit development [10]. *60S RBP* was identified as one of the suitable reference genes to normalize gene expression data in two different grapevine organs (leaves and berries) [3]. Resetic *et al*[30] also placed *60S RBP* in the middle of the ranking order for assessing the average expression stability during olive fruit development.

Both GeNorm and BestKeeper ranked *OUB2*, *TUBA* and *TIP2* as poor performers as reference genes across the three cultivars as a whole. Nonis *et al*[29] has ranked *OUB2* in the middle of the ranking order with an M value of 0.48 while *UBQ10* displayed a higher M value of 0.78 in olive fruit tissues by Resetic *et al*[30]. *UBQ14* was identified as one of the most stable reference gene across different plant organs in cotton [10]. *UBQ10* exhibits very stable expression in *Arabidopsis*[8] however performed poorly as a reference in soybean [9] and in grapevine [17]. *TUBA* displayed a higher M value of 0.80 in olive fruit tissues by Resetic *et al*[30]. *TUBA* was identified as being very stably expressed across development in soybean while was highly unstable across tissues and organs of tomato at different developmental stages [33]. It is interesting to note that though *TIP41* was chosen as one of the best reference genes in olive fruit tissues in the cultivar Istrska belica [30], this reference gene performed poorly in the present study. While *TIP2* outperformed several traditional reference genes in *Arabidopsis* across different tissues, organs and developmental stages [8], it performed poorly in grapevine. This analysis clearly suggested that reference genes are regulated differently in different plant species and/or cultivar [10] and also highlights the importance of validating putative reference genes in different species/tissues/cultivar/conditions.

GeNorm and BestKeeper was further used to evaluate the expression stability of the eight reference genes in each cultivar independently. It was interesting to note that the M values varied dramatically across the cultivars where M values in Barnea samples ranged from 0.9-2.4 while in Frantoio and Picual samples they ranged between 0.5-1.85. In Barnea, *GAPDH*, *EF1-alpha* and *PP2A* were determined to be the three most stable reference genes which is consistent with the analysis performed across the three cultivars together. In Frantoio, slight variations between the two algorithms were observed. Combining data from both the algorithms *GAPDH*, *EF1-alpha* and *60S RBP L18-3* were the top three stable reference genes identified in Frantoio while *60S RBP L18-3, OUB2* and *PP2A* were identified as the top three stable reference genes in Picual. All three cultivars ranked *TIP2* as the least stable gene. Thus it is observed that though few best/worst performing reference genes are common amongst cultivars, their ranking orders do vary and the top ranking genes should be used for their respective cultivars for accurate normalization. This analysis further validated our earlier statement that reference genes may show slightly different expression stability across cultivars.

GeNorm also provides a measure for the optimal number of stable controls that should be used for normalization based on pairwise variation analysis between subsequent normalisation factors. According to GeNorm V, a combination of six most stable reference genes was calculated as being optimal for gene expression studies across the three cultivars when analysed together with the lowest pairwise variation value of 0.18 (Figure 2B). In Barnea, Frantoio and Picual, individually, the lowest pairwise variations were for V5/6, V3/4 and V5/6, respectively (Figure 3B, D, F). According to Vandesompele [11] the optimal cut-off V number should be around 0.15, however many other studies using this application have resulted in higher pairwise variations [7,36-38]. The GeNorm threshold is not a strict cut-off value but it’s an ideal value to provide guidance to researchers to determine the optimal number of reference genes and that the observed trend of changing pairwise variation values is equally informative [9,39]. Depending on the aim of the study, the optimal number of stable controls that should be used for normalization against the target genes should be decided. In case of a comparatively small study, it is impractical to use excessive numbers of reference genes for normalization and thus the minimal use of three most stable reference genes is recommended for calculating the normalization factors [11,31,40]. Therefore *GAPDH*, *EF1-alpha* and *PP2A* were recommended as the three most stable reference genes for normalization against the target genes across the three cultivars together and in the cultivar Barnea. In Frantoio, *GAPDH*, *EF1-alpha* and *60S RBP L18-3* were recommended as the three most stable reference genes while in Picual, *60S RBP L18-3, OUB2* and *PP2A* were recommended as the three most stable reference genes.

## Conclusions

In this study we have investigated the expression of eight candidate reference genes at different developmental stages of the olive fruit across three different olive cultivars independently and together, in an attempt to identify most suitable reference genes for normalizing gene expression across cultivars. *GAPDH*, *EF1-alpha* and *PP2A* were found to be the most stable reference genes in olive mesocarp tissues across the cultivars and in the cultivar, Barnea. *GAPDH*, *EF1-alpha* and *60S RBP L18-3*were found to be most stable reference genes in the cultivar Frantoio while *60S RBP L18-3, OUB2* and *PP2A* were found to be most stable reference genes in the cultivar Picual. In summary, this is one of the first reports on the evaluation of candidate reference genes across three different *O. europaea* cultivars and will provide guidance to other researchers to select reference genes for normalization against target genes by qPCR in this species.

## Methods

### Plant materials

Samples were collected from individual olive trees (*Olea europaea*) of the cultivars Barnea, Frantoio and Picual from Boort, Victoria, Australia during the 2009 crop season. Olive fruits were collected at four different developmental stages [96, 109, 116 and 136 days after flowering (DAF)] during the growth period of the olive and preserved with RNA Later Tissue Collection: RNA stabilization solution (Life Technologies).

### Total RNA isolation and cDNA synthesis

Total RNA was extracted from the mesocarp of olive fruit tissues harvested at four individual timepoints from olive cultivars Barnea, Frantoio and Picual using the RNeasy plant mini kit (Qiagen) according to the supplied protocol. On-column DNase digestion was performed using the RNase-free-DNase set (Qiagen). DNase treated RNA was eluted in RNase free ddH_2_O and stored at -80°C until required. The integrity of all RNA samples were assessed by gel electrophoresis and the quality of the RNA samples were judged by their RNA integrity number (RIN) calculated by the Agilent 2100 Bioanalyzer [41]. In this study only RNA samples with RIN values ≥8 were used for subsequent analysis. For all samples, cDNA was synthesized from 100 ng of total RNA in 20 μL reaction volumes using Thermoscript RT-PCR system (Invitrogen) with oligo-dT primer (50 μM) according to the manufacturer’s protocol. The cDNA samples were diluted 5-fold and a final volume of 10 μL cDNA was used for all real time PCR reactions.

### Selection of candidate reference genes and primer design

A total of eight reference genes were chosen based on their stability in olive tissues as well as in other plants from a publicly available database of olive ESTs [26], where consensus sequences derived from atleast 10 ESTs were chosen for designing primers for the amplification of each of the selected reference genes (Table 1).

Amongst the eight candidate reference genes, two reference genes, *GAPDH* and *PP2A* which performed the best in the Nonis *et al*[29] study were included in this study, although different sequences/contigs were chosen (Table 1). Further, five reference genes common to both the studies were also chosen, namely *EF1-alpha, TIP2, 60S RBP L18-3, TUBA* and *OUB2*. Another novel reference gene, *PTB* identified in the *Arabidopsis thaliana* Affymetrix ATH1 microarray data [8] was also included in the analysis. Primers used for qPCR were designed with Beacon Designer™ software (http://www.premierbiosoft.com) (Table 1).

### Amplification and sequencing of the reference genes

All genes were amplified from the pooled cDNA and were carried out in total volumes of 50 μL, containing 50 ng template cDNA, 1X PCR Buffer, 0.2 mM dNTPs, 1.5 mM MgCl_2_, 0.5 μM each primer (forward and reverse) and 2U Platinum *Taq p*olymerase. The cycling conditions for the amplification of PCR products involved an initial denaturation of 95°C/5 minutes, followed by 35 cycles of 95°C/30s, primer-specific annealing temperature/30s (Table 1) and 72°C/1 minute. The PCR products were visually assessed on 1% agarose gels by electrophoresis and the products were sequenced directly using the ABI Prism BigDye Terminator V3.1 Cycle Sequencing Kit (Applied Biosystems) in a final volume of 20 μL according to a modified version of the standard protocol, containing 1 μL BigDye Premix, 3.5 μL 5X reaction buffer [250 mM Tris HCl (pH 9.0), 10 mM MgCl_2_], 1 μL of forward or reverse primer (3-5 pM) and PCR products (~10 ng). The analysis of the sequencing results was conducted using the BioEdit software package 7.0.9, [http://www.mbio.ncsu.edu/bioedit/bioedit.html] [42] and the identity of the amplified PCR products was confirmed by BLASTn analysis against the non-redundant (nr) GenBank database to confirm the PCR specificity of the primer pairs.

### Preparation of standards and experimental setup of qPCR

Standards for qPCR were used to generate calibration curves to calculate the PCR efficiency of each primer set. For preparation of the standards, cDNA stocks synthesized from each RNA sample were pooled and aliquoted in single-use tubes. Five series of four-fold dilutions were prepared using the undiluted cDNA pool [1:1 (STD1), 1:4 (STD4), 1:16 (STD16), 1:64 (STD64), 1:256 (STD256)]. These dilution series were diluted further in a 1:5 dilution with sterile ddH_2_O and a final volume of 10 μL cDNA was used in each real time PCR reaction. To ensure methodological reproducibility, a total of 12 samples harvested at four individual timepoints from olive cultivars Barnea, Frantoio and Picual were measured in duplicate in a single run, for each gene, where all duplicates were derived from independent RNA extractions and cDNA synthesis reactions. Details of all samples used in the qPCR study are in Additional file 5.

### qRT-PCR methodology

All reactions were performed in 20 μL volumes containing 2 μL of primer mix (5 μM of each forward and reverse primer) (Table 1), 10 μL of 5-fold diluted cDNA (10 ng) and 4 μL SYBR Green I Master mix reagent (Roche) with the LightCycler Carousel-Based system (Roche). The reactions were subjected to an initial denaturation of 72°C/10 minutes, followed by 45 cycles of 95°C/10 seconds, primer-specific annealing/0-10seconds (Table 1) and 72°C/amplicon size (bp)/25 seconds. A melting curve analysis was performed at the end of PCR with a ramp rate of 0.1°C/second. The expression stabilities of the tested genes were validated with two software programs, GeNorm module in qBase Plus software version 2.4 (Biogazelle) and BestKeeper [14].

### GeNorm

Raw Cq values from qPCR were imported into the qBase Plus software package version 2.4 (Biogazelle) and converted to normalised relative quantities (NRQs) using the classic delta-delta Ct method with multiple reference genes [4] to derive fold change gene expression. The average expression stability of the reference genes were analysed using the GeNorm module integrated in qBasePlus. GeNorm is a statistical algorithm which relies on the principle that two ideal reference genes will be equally expressed in all samples irrespective of any experimental condition or tissue type and are minimally regulated [3,7,31]. GeNorm M determines expression stability measure (M value) of all the reference genes under investigation based on the geometric averaging of multiple reference genes and mean pairwise variation of a gene from all other reference genes in a set of samples. Lower M values reflect greater stability of the reference genes. GeNorm M ranks the candidate genes from the most unstable gene to a single most stable gene. GeNorm V calculates the normalisation factor (NF_n_) by calculating the geometric mean of the expression levels of the stable most reference genes by step-wise inclusion of a less stable gene [8,31]. The program calculates the pairwise variation V_n_/V_n+1_ between two sequential normalisation factors, NF_n_ and NF_n+1_. A large variation indicates that the added gene has significant contribution to the normalisation and thus should be included for calculation. If the variation is low (V_n_/V_n+1_ < 0.15) this suggests that the added reference gene is not required for calculation of the normalisation factor and thus can be excluded.

### BestKeeper analysis

The stability of the eight reference genes was also evaluated using the Excel based tool Bestkeeper [14]. BestKeeper ranks the stability of candidate reference genes by performing a statistical analysis of the Cq values based on three variables: Pearson correlation coefficient (r), standard deviation (SD) and percentage covariance (CV). It performs numerous pair-wise correlation analysis of all pairs of candidate reference genes by combining all highly correlated (and putatively stably expressed) reference genes into an index value (BestKeeper index) by calculating the geometric mean. If the reference genes are stably expressed, their expression levels will be highly correlated [14]. The correlation between each candidate reference gene and the index is calculated to determine the relationship between the index and the contributing reference genes by Pearson correlation coefficient (r), coefficient of determination (*r*^2^) and the probability p values.

## Abbreviations

cDNA: Complementary DNA; CV: Coefficient of variance; DAF: Days after flowering; RT-qPCR: Quantitative real-time reverse transcriptase polymerase chain reaction; SE: Standard error; SD: Standard deviation; 60S RBP L18-3: 60S ribosomal protein L18; PP2A: Serine/threonine protein phosphatase 2A; PTB: Polypyrimidine tract-binding protein; TUBA: Tubulin alpha; TIP2: Aquaporin tonoplast intrinsic protein; OUB2: Polyubiquitin; GAPDH: Glyceraldehyde 3-phosphate dehydrogenase; EF1-α: Elongation factor 1 alpha.

## Competing interests

The authors declare that they have no competing interests.

## Authors’ contributions

DLR and JCJ designed the project. DLR performed the experimental procedures, data analysis and drafted the manuscript. JCJ supervised the study and critically revised the manuscript. Both authors read and approved the final manuscript.

## Supplementary Material

Additional file 1 **Agarose gel electrophoresis of PCR products of eight reference genes.** Lanes: M: 100bp molecular weight marker; Lane1: glyceraldehyde 3-phosphate dehydrogenase, Lane2: 60S ribosomal protein L18-3, Lane 3: serine/threonine protein phosphatase 2A, Lane 4: polypyrimidine tract-binding protein, Lane5: tubulin alpha, Lane6: aquaporin tonoplast intrinsic protein, Lane 7: polyubiquitin, Lane8: elongation factor 1 alpha, Lane 9: water-only negative control. Click here for file

Additional file 2 **Representative efficiency curves for individual reference genes. Mean Cq values were plotted against the five four-fold cDNA serial dilutions (1:1, 1:4, 1:16, 1:64, 1:256) using the qBase Plus software.** Slope obtained for each plot has been shown top right. Click here for file

Additional file 3 **Melting curve peak of eight candidate reference genes.** Fluorescence values were plotted against temperature (˚C) using the Light cycler Carousel (Roche). Click here for file

Additional file 4 **The transcription profiles of individual reference genes given as Cq values across all samples in Barnea, Frantoio and Picual.** Average Cq values with the standard deviation (SD) for all samples shown. Repli: Replicate. Annotation for each sample with their name of olive cultivar, timepoint and year has been given in Additional file 5. Click here for file

Additional file 5 **Annotations for all olive cDNA samples, standards and negative controls used in the qPCR study.** A. Annotations for olive cDNA samples. B. Annotations for standards and negative controls. Click here for file
