# Supplementary figures and images for: Validation of reference genes for gene expression analysis in olive (Olea europaea) mesocarp tissue by quantitative real-time RT-PCR
Source: BMC Res Notes. 2014 May 18;7:304. doi: 10.1186/1756-0500-7-304 (PMC4062307; doi:10.1186/1756-0500-7-304)

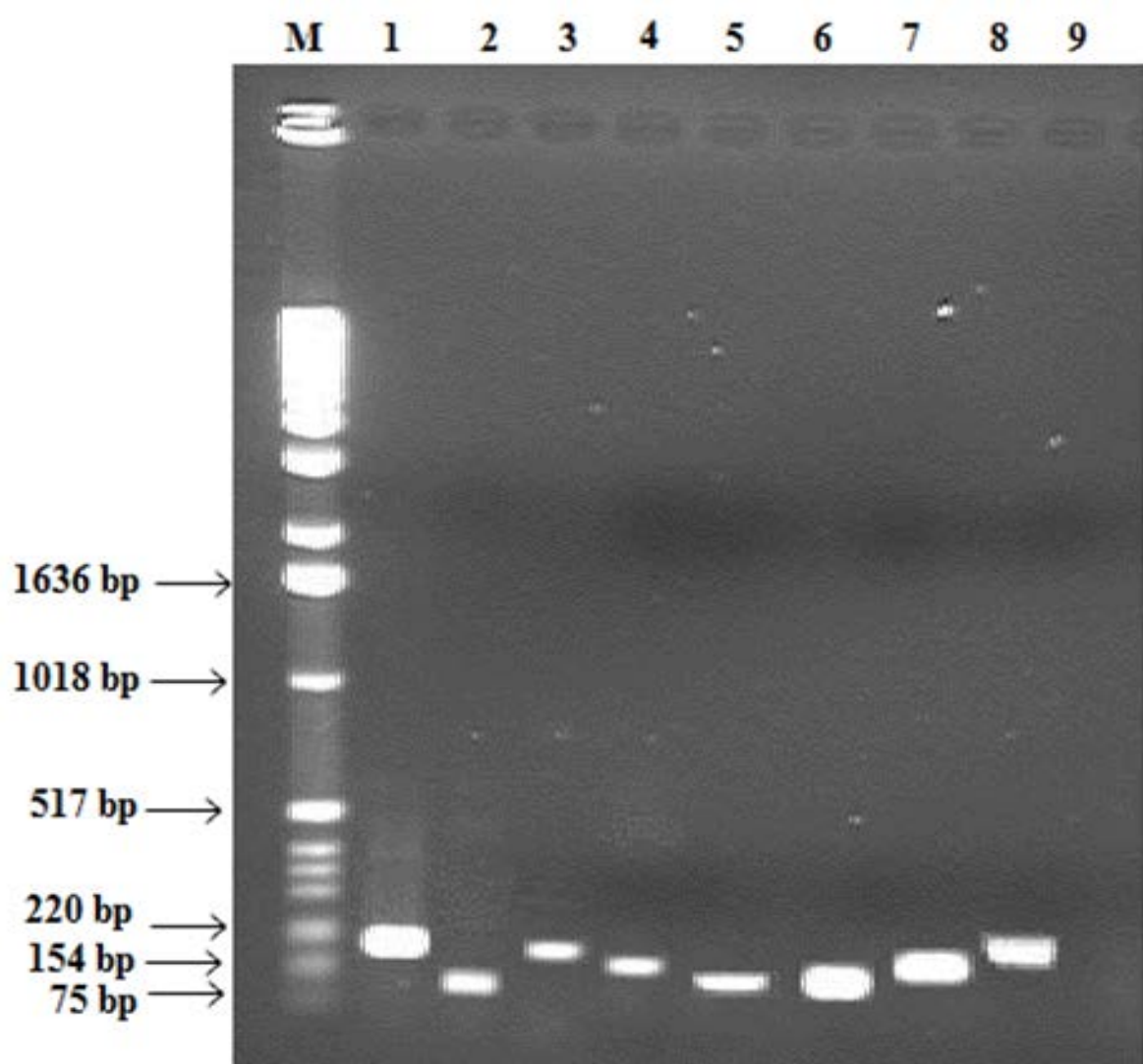

Supplement: Additional file 1 — Agarose gel electrophoresis of PCR products of eight reference genes. Lanes: M: 100bp molecular weight marker; Lane1: glyceraldehyde 3-phosphate dehydrogenase, Lane2: 60S ribosomal protein L18-3, Lane 3: serine/threonine protein phosphatase 2A, Lane 4: polypyrimidine tract-binding protein, Lane5: tubulin alpha, Lane6: aquaporin tonoplast intrinsic protein, Lane 7: polyubiquitin, Lane8: elongation factor 1 alpha, Lane 9: water-only negative control. [file 1756-0500-7-304-S1.pdf]

**60S RBP L18-3**

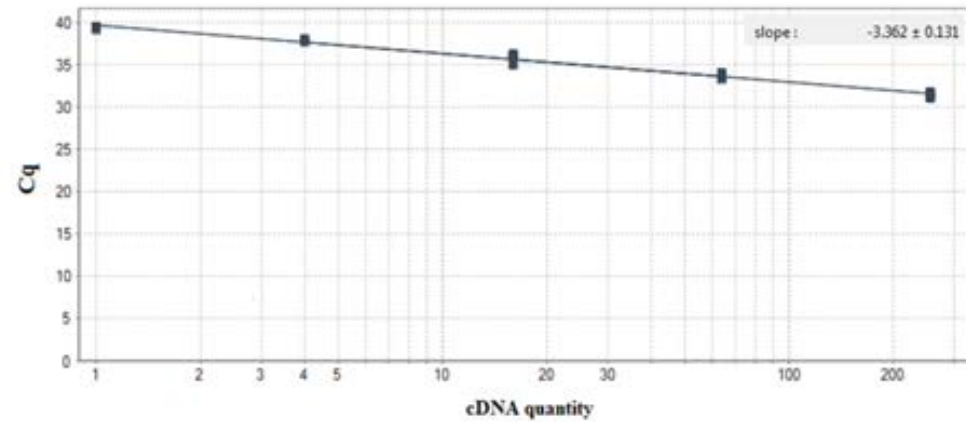

**EF1-alpha**

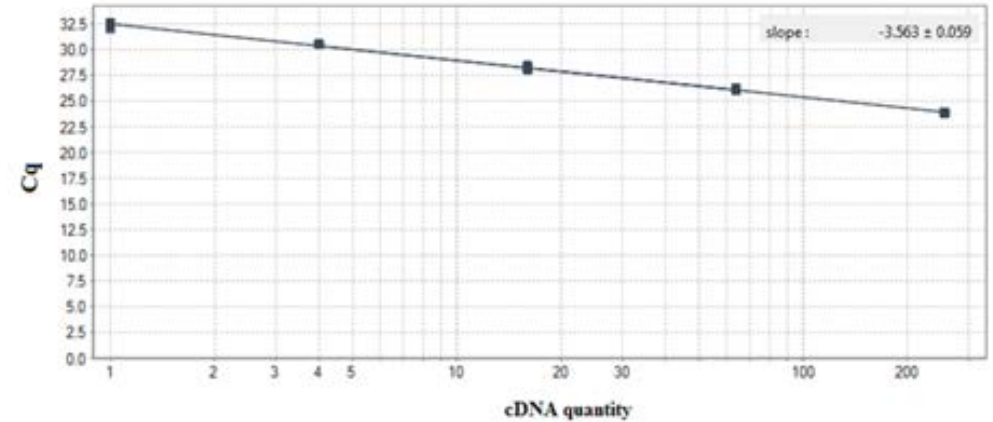

**PP2A**

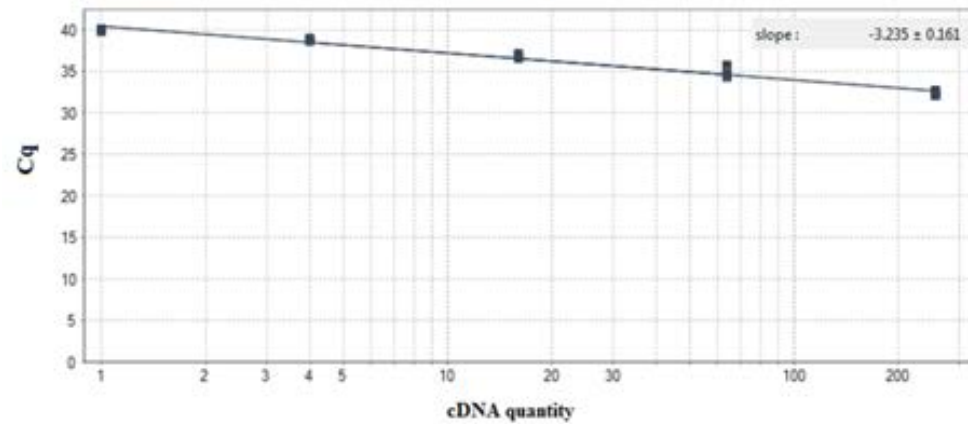

**GAPDH**

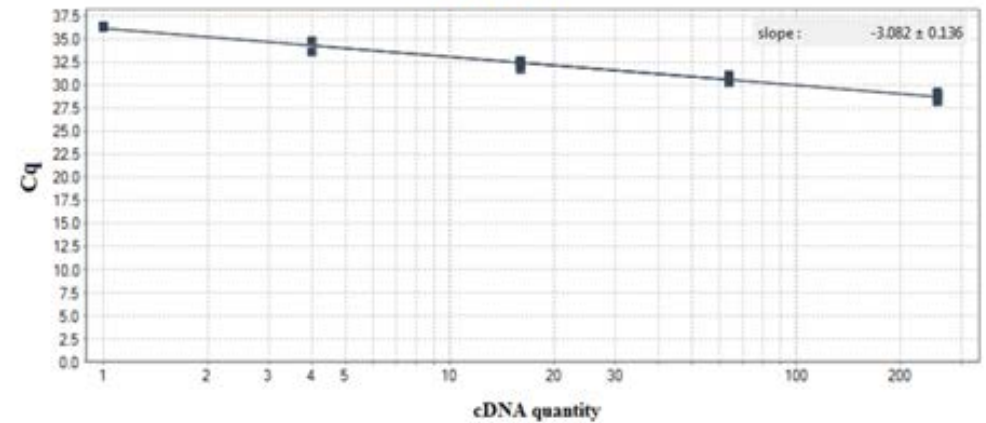

**PTB**

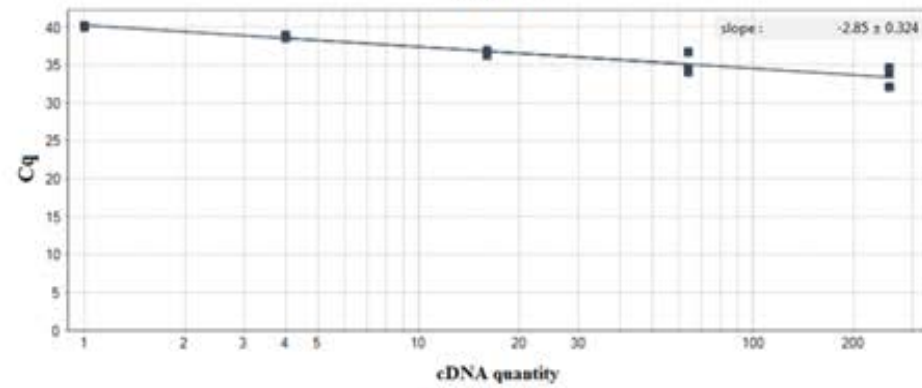

**TIP2**

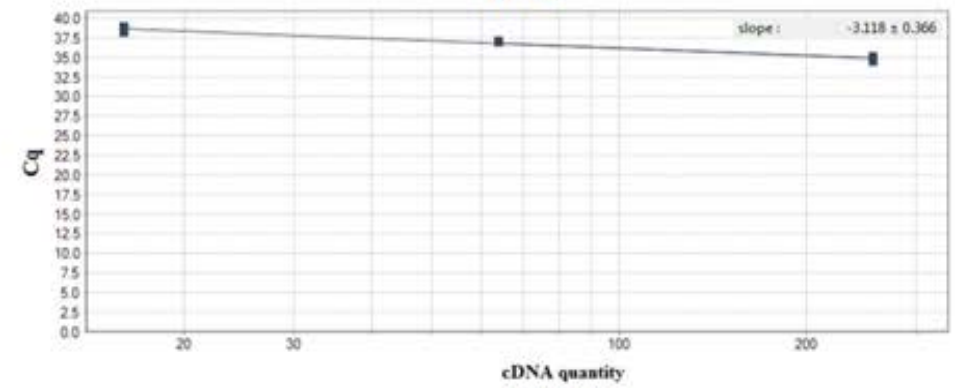

**TUBA**

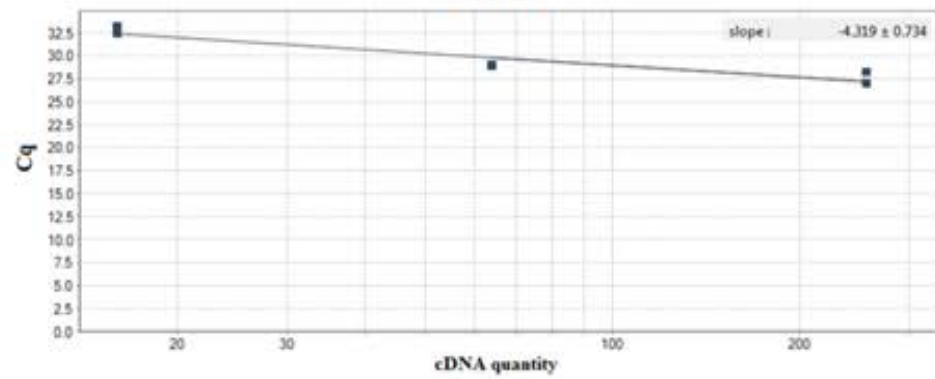

**OUB2**

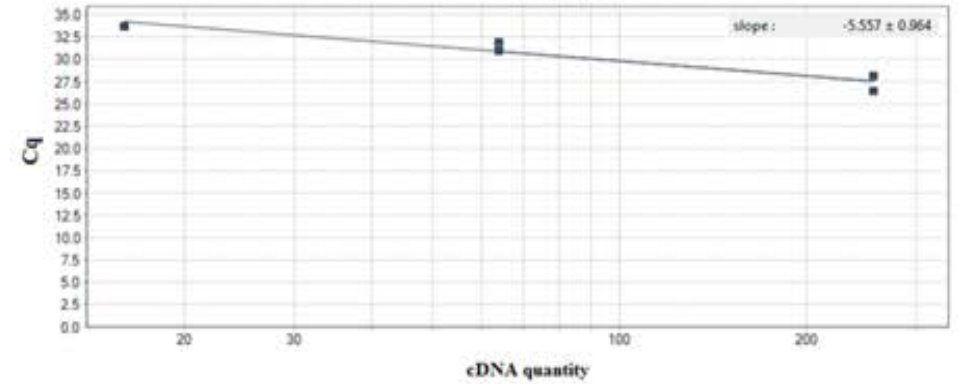

Supplement: Additional file 2 — Representative efficiency curves for individual reference genes. Mean Cq values were plotted against the five four-fold cDNA serial dilutions (1:1, 1:4, 1:16, 1:64, 1:256) using the qBase Plus software. Slope obtained for each plot has been shown top right. [file 1756-0500-7-304-S2.pdf]

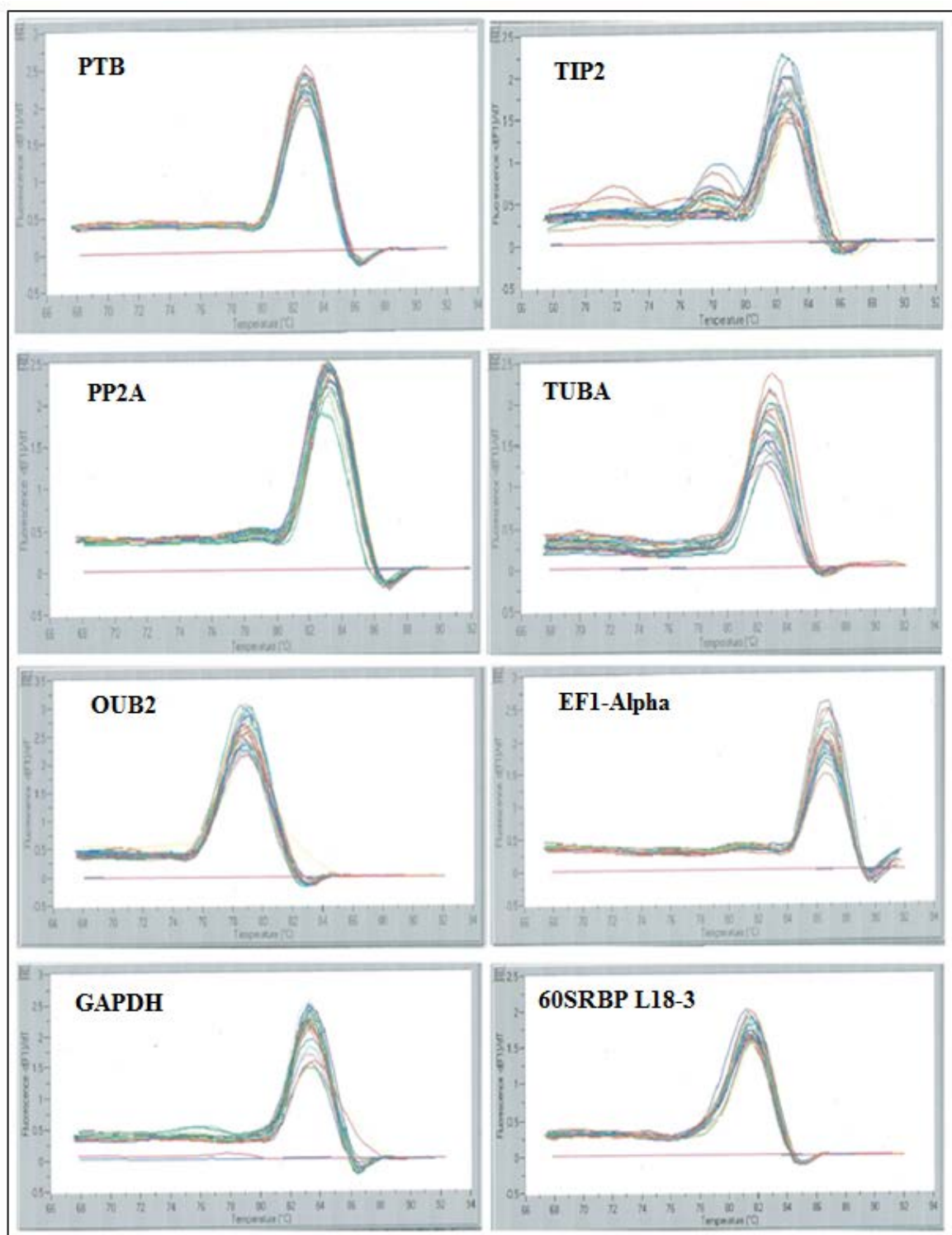

Supplement: Additional file 3 — Melting curve peak of eight candidate reference genes. Fluorescence values were plotted against temperature (˚C) using the Light cycler Carousel (Roche). [file 1756-0500-7-304-S3.pdf]
