# Supplementary material for: Validation of reference genes for gene expression analysis in olive (Olea europaea) mesocarp tissue by quantitative real-time RT-PCR
Source: BMC Res Notes. 2014 May 18;7:304. doi: 10.1186/1756-0500-7-304 (PMC4062307; doi:10.1186/1756-0500-7-304)
Supplement: Additional file 4 — The transcription profiles of individual reference genes given as Cq values across all samples in Barnea, Frantoio and Picual. Average Cq values with the standard deviation (SD) for all samples shown. Repli: Replicate. Annotation for each sample with their name of olive cultivar, timepoint and year has been given in Additional file 5. [file 1756-0500-7-304-S4.docx]

**Additional file 4. The transcription profiles of individual reference genes given as Cq values across all samples in Barnea, Frantoio and Picual**

|  |  | ***60S RBP L18-3*** |  |  | ***PP2A*** |  |  | ***EFI-alpha*** |  |  | ***GAPDH*** |  |
| --- | --- | --- | --- | --- | --- | --- | --- | --- | --- | --- | --- | --- |
| **Samples** | **Cq value** | **Average Cq** | **SD** | **Cq value** | **Average Cq** | **SD** | **Cq value** | **Average Cq** | **SD** | **Cq value** | **Average Cq** | **SD** |
| **B-1-09** | 28.89 | 29.01 | 0.17 | 29.51 | 29.47 | 0.05 | 21.41 | 21.61 | 0.28 | 26.99 | 27.04 | 0.07 |
| **Repli. of B-1-09** | 29.14 |  |  | 29.43 |  |  | 21.82 |  |  | 27.09 |  |  |
| **B-2-09** | 31.24 | 31.14 | 0.14 | 35.50 | 34.91 | 0.82 | 24.76 | 24.74 | 0.02 | 30.60 | 30.50 | 0.13 |
| **Repli. of B-2-09** | 31.04 |  |  | 34.33 |  |  | 24.72 |  |  | 30.41 |  |  |
| **B-3-09** | 30.32 | 30.17 | 0.20 | 31.43 | 31.26 | 0.24 | 22.26 | 22.34 | 0.11 | 27.56 | 27.65 | 0.13 |
| **Repli. of B-3-09** | 30.03 |  |  | 31.09 |  |  | 22.42 |  |  | 27.75 |  |  |
| **B-4-09** | 33.86 | 34.04 | 0.25 | 33.06 | 33.58 | 0.73 | 24.44 | 24.43 | 0.01 | 28.07 | 28.28 | 0.30 |
| **Repli. of B-4-09** | 34.22 |  |  | 34.10 |  |  | 24.42 |  |  | 28.50 |  |  |
| **F-1-09** | 30.69 | 30.71 | 0.02 | 32.16 | 32.06 | 0.14 | 21.97 | 21.93 | 0.04 | 27.85 | 27.80 | 0.06 |
| **Repli. of F-1-09** | 30.73 |  |  | 31.96 |  |  | 21.90 |  |  | 27.76 |  |  |
| **F-2-09** | 32.15 | 32.18 | 0.04 | 32.12 | 32.37 | 0.35 | 23.15 | 23.17 | 0.02 | 28.03 | 28.13 | 0.14 |
| **Repli. of F-2-09** | 32.22 |  |  | 32.62 |  |  | 23.19 |  |  | 28.23 |  |  |
| **F-3-09** | 31.98 | 31.96 | 0.02 | 32.97 | 32.94 | 0.03 | 22.81 | 22.81 | 0.00 | 29.23 | 29.40 | 0.24 |
| **Repli. of F-3-09** | 31.95 |  |  | 32.92 |  |  | 22.82 |  |  | 29.58 |  |  |
| **F-4-09** | 31.46 | 31.31 | 0.20 | 34.24 | 34.46 | 0.31 | 23.59 | 23.11 | 0.67 | 29.33 | 29.23 | 0.13 |
| **Repli. of F-4-09** | 31.17 |  |  | 34.69 |  |  | 22.63 |  |  | 29.14 |  |  |
| **P-1-09** | 31.10 | 31.06 | 0.04 | 31.60 | 31.66 | 0.09 | 22.93 | 22.88 | 0.07 | 27.59 | 27.65 | 0.09 |
| **Repli. of P-1-09** | 31.03 |  |  | 31.73 |  |  | 22.83 |  |  | 27.72 |  |  |
| **P-2-09** | 32.20 | 32.09 | 0.14 | 32.42 | 32.35 | 0.09 | 24.57 | 24.55 | 0.02 | 28.27 | 28.56 | 0.41 |
| **Repli. of P-2-09** | 31.99 |  |  | 32.29 |  |  | 24.54 |  |  | 28.86 |  |  |
| **P-3-09** | 31.79 | 31.86 | 0.09 | 32.53 | 32.53 | 0.00 | 22.74 | 22.76 | 0.03 | 28.03 | 28.09 | 0.09 |
| **Repli. of P-3-09** | 31.93 |  |  | 32.54 |  |  | 22.79 |  |  | 28.16 |  |  |
| **P-4-09** | 32.09 | 32.12 | 0.04 | 33.54 | 33.60 | 0.09 | 25.17 | 25.31 | 0.19 | 30.86 | 30.73 | 0.17 |
| **Repli. of P-4-09** | 32.15 |  |  | 33.67 |  |  | 25.45 |  |  | 30.61 |  |  |

Average Cq values with the standard deviation (SD) for all samples shown

Repli: Replicate

Annotation for each sample with their name of olive variety, timepoint and year has been given in Additional file 5.

|  |  | ***OUB2*** |  |  | ***TIP2*** |  |  | ***PTB*** |  |  | ***TUBA*** |  |
| --- | --- | --- | --- | --- | --- | --- | --- | --- | --- | --- | --- | --- |
| **Samples** | **Cq value** | **Average Cq** | **SD** | **Cq value** | **Average Cq** | **SD** | **Cq value** | **Average Cq** | **SD** | **Cq value** | **Average Cq** | **SD** |
| **B-1-09** | 23.03 | 23.48 | 0.64 | 26.87 | 27.54 | 0.94 | 32.37 | 32.27 | 0.14 | 27.51 | 27.49 | 0.02 |
| **Repli. of B-1-09** | 23.94 |  |  | 28.21 |  |  | 32.17 |  |  | 27.47 |  |  |
| **B-2-09** | 26.28 | 25.70 | 0.81 | 35.97 | 36.07 | 0.14 | 37.63 | 37.42 | 0.29 | 30.47 | 30.45 | 0.02 |
| **Repli. of B-2-09** | 25.13 |  |  | 36.17 |  |  | 37.21 |  |  | 30.44 |  |  |
| **B-3-09** | 25.88 | 26.28 | 0.56 | 39.31 | 38.82 | 0.69 | 32.63 | 33.15 | 0.73 | 33.04 | 32.50 | 0.76 |
| **Repli. of B-3-09** | 26.68 |  |  | 38.33 |  |  | 33.67 |  |  | 31.96 |  |  |
| **B-4-09** | 23.94 | 24.07 | 0.19 | 35.86 | 35.69 | 0.23 | 37.04 | 37.20 | 0.22 | 31.34 | 31.42 | 0.12 |
| **Repli. of B-4-09** | 24.21 |  |  | 35.53 |  |  | 37.36 |  |  | 31.51 |  |  |
| **F-1-09** | 24.12 | 24.04 | 0.11 | 35.12 | 35.31 | 0.26 | 34.64 | 34.37 | 0.37 | 31.19 | 30.95 | 0.33 |
| **Repli. of F-1-09** | 23.96 |  |  | 35.50 |  |  | 34.11 |  |  | 30.71 |  |  |
| **F-2-09** | 23.25 | 23.41 | 0.22 | 34.63 | 34.47 | 0.22 | 34.58 | 34.35 | 0.31 | 28.13 | 27.70 | 0.60 |
| **Repli. of F-2-09** | 23.57 |  |  | 34.31 |  |  | 34.13 |  |  | 27.27 |  |  |
| **F-3-09** | 23.45 | 23.68 | 0.33 | 36.21 | 36.16 | 0.07 | 35.15 | 34.87 | 0.38 | 29.83 | 30.12 | 0.41 |
| **Repli. of F-3-09** | 23.92 |  |  | 36.11 |  |  | 34.60 |  |  | 30.41 |  |  |
| **F-4-09** | 24.21 | 23.94 | 0.37 | 32.21 | 32.44 | 0.33 | 34.19 | 34.34 | 0.21 | 32.14 | 32.22 | 0.11 |
| **Repli. of F-4-09** | 23.68 |  |  | 32.68 |  |  | 34.50 |  |  | 32.30 |  |  |
| **P-1-09** | 21.80 | 21.73 | 0.09 | 27.55 | 27.67 | 0.16 | 33.71 | 33.38 | 0.46 | 28.20 | 28.30 | 0.14 |
| **Repli. of P-1-09** | 21.67 |  |  | 27.79 |  |  | 33.05 |  |  | 28.40 |  |  |
| **P-2-09** | 25.92 | 26.20 | 0.40 | 38.92 | 38.45 | 0.66 | 34.84 | 34.68 | 0.22 | 29.15 | 28.99 | 0.22 |
| **Repli. of P-2-09** | 26.49 |  |  | 37.98 |  |  | 34.52 |  |  | 28.83 |  |  |
| **P-3-09** | 24.22 | 24.40 | 0.25 | 35.42 | 35.66 | 0.33 | 36.16 | 36.09 | 0.09 | 29.70 | 29.58 | 0.16 |
| **Repli. of P-3-09** | 24.58 |  |  | 35.90 |  |  | 36.02 |  |  | 29.46 |  |  |
| **P-4-09** | 25.94 | 25.73 | 0.29 | 35.33 | 35.42 | 0.12 | 34.83 | 35.26 | 0.60 | 32.69 | 32.98 | 0.41 |
| **Repli. of P-4-09** | 25.52 |  |  | 35.51 |  |  | 35.69 |  |  | 33.27 |  |  |

Average Cq values with the standard deviation (SD) for all samples shown

Repli: Replicate

Annotation for each sample with their name of olive variety, timepoint and year has been given in Additional file 5.
