# Supplementary material for: Validation of reference genes for gene expression analysis in olive (Olea europaea) mesocarp tissue by quantitative real-time RT-PCR
Source: BMC Res Notes. 2014 May 18;7:304. doi: 10.1186/1756-0500-7-304 (PMC4062307; doi:10.1186/1756-0500-7-304)
Supplement: Additional file 5 — Annotations for all olive cDNA samples, standards and negative controls used in the qPCR study. A. Annotations for olive cDNA samples. B. Annotations for standards and negative controls. [file 1756-0500-7-304-S5.docx]

**Additional file 5. Annotations for all olive cDNA samples, standards and negative controls used in the qPCR study**


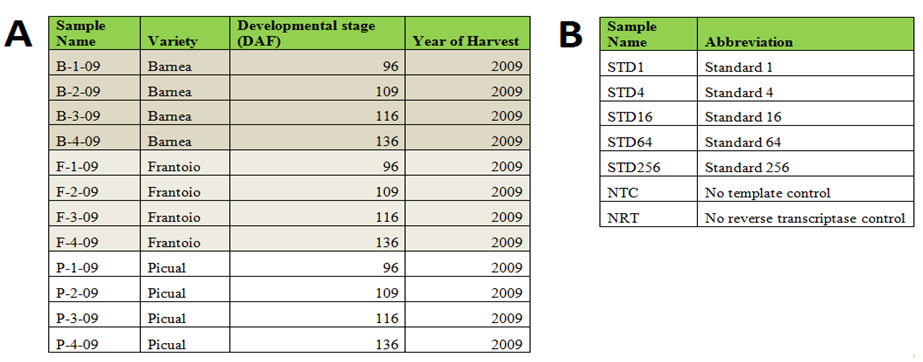


1. Annotations for olive cDNA samples
2. Annotations for standards and negative controls
